# Correction to ‘Natural Frequency Method: estimating the preferred walking speed of *Tyrannosaurus rex* based on tail natural frequency’

**DOI:** 10.1098/rsos.211139

**Published:** 2021-07-21

**Authors:** Pasha A. van Bijlert, A. J. ‘Knoek’ van Soest, Anne S. Schulp

*R. Soc. Open Sci.***8**, 201441. (Published online 21 April 2021) (doi:10.1098/rsos.201441)

This correction refers to the caption for figure 2. An incorrect reference was given; the correct reference is [39]. This has now been corrected.

